# Fast quantitative bone marrow lesion measurement on knee MRI for the assessment of osteoarthritis

**DOI:** 10.1016/j.ocarto.2022.100234

**Published:** 2022-01-10

**Authors:** Frank Preiswerk, Meera S. Sury, Jeremy R. Wortman, Gesa Neumann, William Wells, Jeffrey Duryea

**Affiliations:** a Department of Radiology, Brigham and Women's Hospital, Harvard Medical School, Boston, MA, 02115, USA; b Department of Radiology, Lahey Hospital and Medical Center, Tufts University School of Medicine, Burlington, MA, 01805, USA; c Department of Radiology, Boston Medical Center, Boston University, Boston, MA, 02118, USA

**Keywords:** Knee osteoarthritis, Bone marrow lesions, MRI, Software assessment

## Abstract

**Objective:**

Knee osteoarthritis (KOA) is a prevalent disease with a high economic and social cost. Magnetic resonance imaging (MRI) can be used to visualize many KOA-related structures including bone marrow lesions (BMLs), which are associated with OA pain. Several semi-automated software methods have been developed to segment BMLs, using manual, labor-intensive methods, which can be costly for large clinical trials and other studies of KOA. The goal of our study was to develop and validate a more efficient method to quantify BML volume on knee MRI scans.

**Materials and methods:**

We have applied a deep learning approach using a patch-based convolutional neural network (CNN) which was trained using 673 MRI data sets and the segmented BML masks obtained from a trained reader. Given the location of a BML provided by the reader, the network performed a fully automated segmentation of the BML, removing the need for tedious manual delineation. Accuracy was quantified using the Pearson's correlation coefficient, by a comparison to a second expert reader, and using the Dice Similarity Score (DSC).

**Results:**

The Pearson's R^2^ value was 0.94 and we found similar agreement when comparing two readers (R^2^ ​= ​0.85) and each reader versus the DL model (R^2^ ​= ​0.95 and R^2^ ​= ​0.81). The average DSC was 0.70.

**Conclusions:**

We developed and validated a deep learning-based method to segment BMLs on knee MRI data sets. This has the potential to be a valuable tool for future large studies of KOA.

## 1 Introduction

Osteoarthritis (OA) is a highly prevalent, painful, and severely debilitating disease with a substantial economic and social impact [1,2]. OA can affect many anatomical locations, but generally has the largest impact on the weight-bearing joints, including the hip and knee. Currently there are no proven disease-modifying OA drugs (DMOADS) [3], although a great deal of effort is underway to develop and test potential therapies [4]. Given the absence of effective treatments to slow or reverse OA, joint replacement is the definitive therapeutic management. In 2014, more than 700,000 individuals in the United Stated underwent knee arthroplasty [5].

Clinical trials and other studies of knee OA (KOA) require objective and efficient methods to test treatment efficacy, and to study the natural history and epidemiology of the disease. Assessments from radiological imaging can provide ideal surrogate outcome measures since they are based on the visualization of KOA-related structures. Conventional radiography and magnetic resonance imaging (MRI) are the most common modalities used for KOA assessment, although ultrasound [6] and computed tomography [7] have seen limited use. Radiography is advantageous due to the cost and convenience, though many of the crucial soft-tissue structures relevant for KOA are invisible radiographically. For example, cartilage loss is inferred indirectly on a radiograph by observing the inter-bone joint space loss. In contrast, MRI can be used to assess soft tissue changes directly and, as a three-dimensional modality, it is ideal for appreciating the complex structure of the knee joint. KOA features including cartilage, bone marrow lesions (BMLs), meniscal damage, osteophytes, and effusion/synovitis are well-visualized on MRI.

MRI assessment of KOA for research studies generally uses one of two approaches: semi-quantitative scoring systems and fully quantitative software processing. Semi-quantitative scoring [8] uses atlas-based systems, assigning ordinal scores for a variety of structures. While such systems are widely used and proven effective, they are time-consuming and fundamentally based on a subjective assessment. Fully quantitative methods using image processing software offer objective and direct measures of the size, shape, and intensity profile of KOA-related structures [[9], [10], [11], [12]]. Unlike the ordinal scales offered by semi-quantitative scoring, the output of quantitative software techniques consists of floating-point numbers, which is ideal for statistical analysis.

Since clinical trials and other studies of KOA often involve thousands of subjects and multiple time points, scans requiring assessment can number in the thousands. Software approaches generally offer time savings compared to semi-quantitative scoring, but can still require substantial reader time. For example, a method to assess BMLs that requires an average of 5 ​min per scan [9] would need 250 ​h of reader time for a study of bilateral KOA with 500 subjects followed over 3 time points (500 ​× ​2 x 3 ​× ​5 ​min).

BMLs, a form of subchondral inflammation, are seen on knee MRI as hyperintense structures in the bone, close to the bone-cartilage interface. BMLs are highly relevant to KOA research since they have been shown to have a stronger association with knee pain than other structures [13]. Unlike cartilage loss, BMLs do not progress unidirectionally over time; studies show a waxing and waning pattern of BML size at subsequent time points [14]. For this reason, responsiveness to change is not an appropriate metric to test the performance of methods measuring BML volume.

Deep learning (DL), the subset of machine learning focused on training deep artificial neural networks, has made considerable advances over the last few years due to the availability of unprecedented computing power through graphical processing units (GPUs), large datasets, and a thriving open-source software ecosystem [15]. In particular, convolutional neural networks (CNNs) have become the state of the art for image classification and segmentation. Studies have used DL methods to segment KOA-related structures on MRI [16,17], but to our knowledge, no published methods have been reported applying DL to measure BML volume. The goal of this study was to develop and validate a DL-based software method to automatically segment KOA-related BMLs with minimal input and substantially decrease reader time for this measurement.

Our previous approach to BML segmentation was two-fold [9]. In the first step, a reader (JW) identified BMLs in each image slice with a single mouse click. The second, more laborious task had a reader (MS) semi-automatically segment each of the identified BMLs using a variable thresholding algorithm. The average total reader time was approximately 5 ​min per scan; on average, less than 30 ​s was required for the first step. The goal of the method reported here was to eliminate the second step while maintaining reliance on the initial BML identification performed by a human reader. The possibility of eliminating the second step would offer considerable savings in time and cost. We hypothesized that a DL-based method to measure BML volume would perform well enough to replace the semi-automated thresholding-based algorithm.

## 2 Materials and Methods

### 2.1 Imaging subjects

We used MRI scans from the Osteoarthritis Initiative, (OAI) a National Institutes of Health (NIH) and industry sponsored study of KOA [18]. The OAI image data are publicly available, fully anonymized, and include 4796 enrolled subjects followed over seven time points: baseline (BL), 12 months, 24 months, 36 months, 48 months, 72 months, and 96 months. To train and test our DL software, we used the BL, 12-month, and 24-month data from the 600 subjects that comprise the OA Biomarkers Consortium FNIH Project, a nested sub-study of the OAI. A single indexed knee was assessed for each subject. Details of this project are provided elsewhere [19]. Our semi-automated method [9] was used to segment BMLs on these data to provide training and test data.

The MRI data were acquired on a Siemens 3T scanners (Trio, Siemens, Erlangen, Germany) using the following protocol: Sagittal turbo spin echo fat-suppressed (TSE FS) (0.357 ​× ​0.357 ​× ​3.0 ​mm^3^, Repetition Time (TR) 3200 ​ms. Echo Time (TE) 30 ​ms.). All 600 subjects had both BL and 24 month scans; 582 were also imaged at the 12 month time point. Therefore, the dataset contained 2–3 scans for each of the 600 subjects, totaling 1782 scans. Selecting only subjects with a BML in at least one time point gave 1358 scans from 544 different subjects for further processing. The 56 subjects with no BMLs at any time point were excluded. The 544 subjects were randomly split into training, validation and test sets of 50% (673 scans, 272 subjects), 25% (352 scans, 186 subjects), 25% (333 scans, 186 subjects), respectively. Since we used data from three OAI time points, there was generally more than one scan per knee.

96 ​× ​96 pixel “patches” that contained the BMLs were extracted using the initial reader marks. Through this process, a dataset of N ​= ​11,676 BML image and mask patch pairs (5673 in training set, 2875 in validation set, 3128 in test set) was obtained for further processing by our CNN. Intensity values alone do not provide sufficient information for an algorithm to correctly segment BMLs, as they have similar gray scale intensity values as the surrounding structures including cartilage and fluid. Much of the manual effort for the semi-automated method is devoted to separating the true BML voxels from adjacent hyperintense tissues, often the most time-consuming component of the semi-automated process. BMLs appear in characteristic locations, often close to cartilage, and CNNs are a powerful method that can take such contextual information into account. Furthermore, there is no need to manually specify these areas, as CNNs automatically learn important features during training.

### 2.2 Model architecture and training

**Fig. 1:**
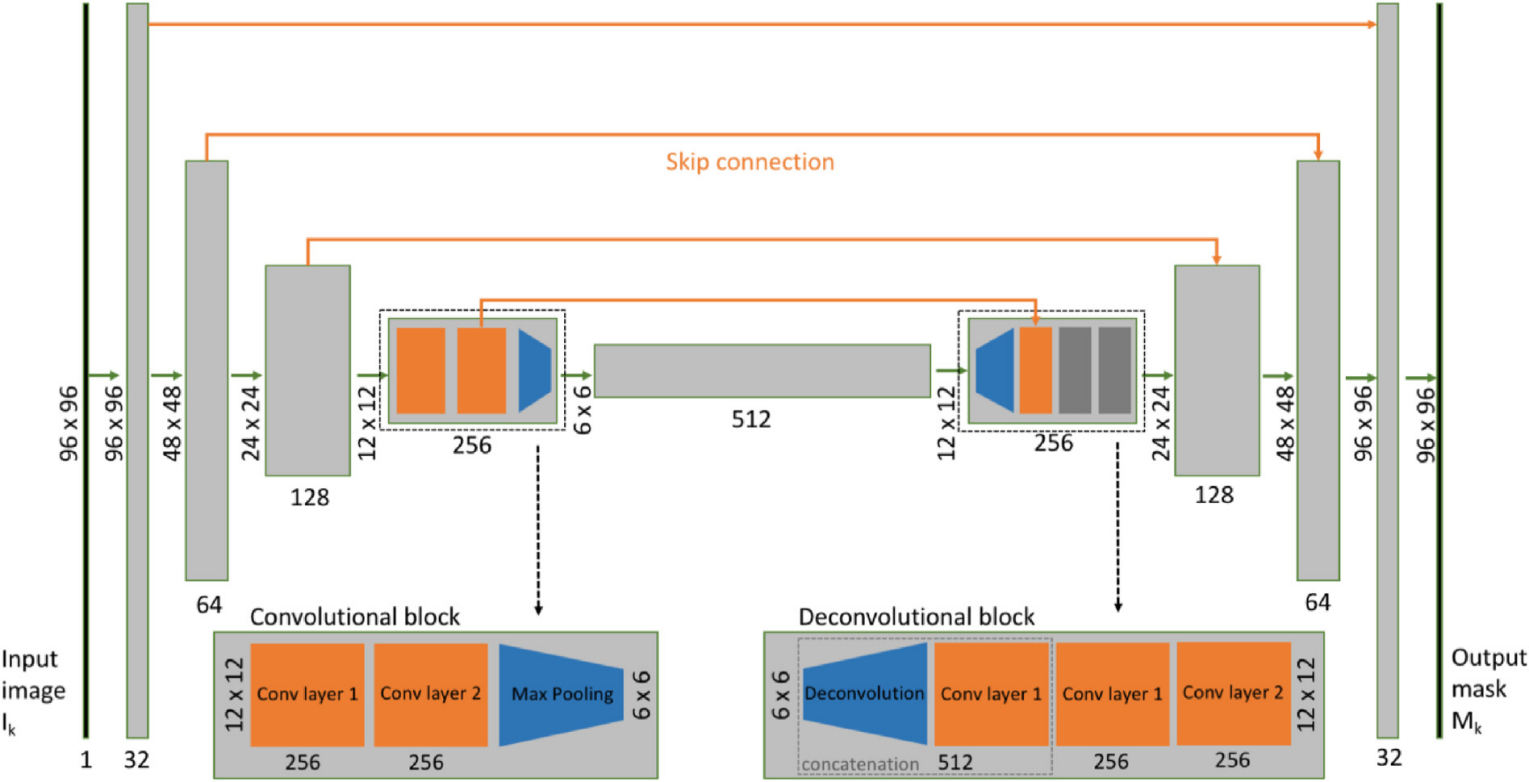
Overview of the U-Net architecture used (5-level version depicted here).

A U-Net convolutional neural network [20] (Fig. 1.) was trained on BML patches and corresponding segmentation mask pairs. The U-Net model is a fully-convolutional network (FCN) architecture [21] specifically designed for image segmentation tasks. FCNs are similar to CNNs but lack the final set of fully connected layers on the output normally used to learn a scalar- or vector-valued decision function over the extracted features. This architecture allows for end-to-end training of image segmentation models.

The U-Net architecture consists of a series of convolution and so-called “max-pooling” layer blocks, which gradually reduce spatial resolution by a factor of two while doubling the number of output channels, eventually reaching a spatially compressed intermediate representation of the input. The process is followed by its deconvolutional counterpart, which decodes the intermediate representation back into the shape of the original image size as depicted in Fig. 1. “Skip connections” between corresponding layers from the encoding and decoding arms support the propagation of high-resolution information, thus avoiding loss of information from the compression process. Fig. 1 depicts how the input of size 96 ​× ​96 pixels changes shape as it passes through the network. In the first resolution block, the two-dimensional convolution layers enhance the number of channels from 1 (grayscales) to 32, and the immediately following max pooling layer reduces the spatial extent by a factor of 2. The resulting 48 ​× ​48 x 32 tensor is passed on to the next resolution block, which performs a similar transformation. The example in Fig. 1 has 5 resolution levels and therefore the tensor size at the deepest level is 6 ​× ​6 x 512. Increasing the number of layers can potentially produce a substantially higher performing CNN, but will increase computational cost and, more importantly, lead to overfitting as the number of model parameters increases with the number of layers and consequently, a CNN that actually has reduced performance on the test set.

The Dice similarity coefficient was used as the loss function [22]. Compared to the more common binary cross-entropy loss, the Dice loss is less sensitive to the class imbalance observed in BML segmentation where the number of foreground pixels is often significantly smaller than the number of background pixels.

The loss was minimized using the Adam stochastic gradient descent scheme [23] (learning rate ​= ​0.00001, $\beta_{1}=0.9$, $\beta_{2}=0.999$) on batches of size 64. All image patches were normalized based on the mean and standard deviation of images in the training set. An NVIDIA Titan Xp graphics processing unit (GPU) (NVIDIA Corporation, Santa Clara, CA) was used for accelerated training. Early-stopping was employed when the validation error did not improve over 20 epochs. All experiments were implemented in Python using Keras [24] with the Tensorflow [25] backend.

### 2.3 Statistical analysis

The main test of performance was to demonstrate accuracy of the DL method compared to the existing segmentation method, which was done graphically and by calculating the Pearson's R^2^ value on a per scan basis. Comparisons between the DL and semi-automated BMLs were also assessed using the Dice Similarity Score (DSC) [22] for individual masks. DSC is a measure of overlap between two ROIs where a DSC ​= ​1 means perfect overlap and DSC ​= ​0 is no overlap. To study the relationship between model capacity and performance on the test set, variations of the above U-Net were assessed with convolution levels ranging from 2 to 6. As stated above, the main goal of applying DL was to eliminate the more time-consuming reader task. To directly test this objective, we used a second reader (GN) to segment a randomly selected subset of 25 scans from the test set using the same marked BML locations as did Reader 1 and the DL algorithm. We hypothesized that the DL to reader agreement would be similar to that of Reader 1 versus Reader 2.

## 3 Results

**Fig. 2:**
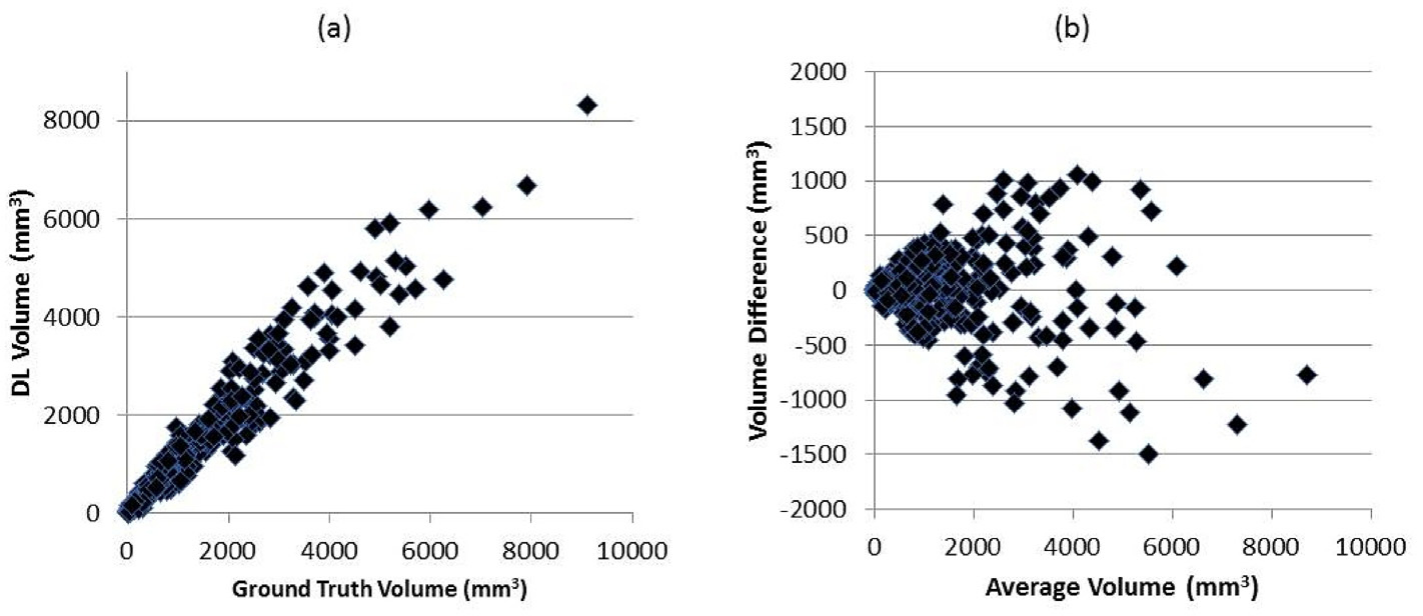
(a) Scatter and (b) Bland-Altman plots of BML volumes of all 333 subjects in the test set.

There was excellent agreement between the DL and semi-automated segmented BMLs as shown in Fig. 2, scatter and Bland-Altman plots of the BML data from the 333 scans in the test group,. Furthermore, the Pearson's R^2^ value was 0.94 and there is no obvious evidence of a bias. The average DSC was 0.7.

**Fig. 3:**
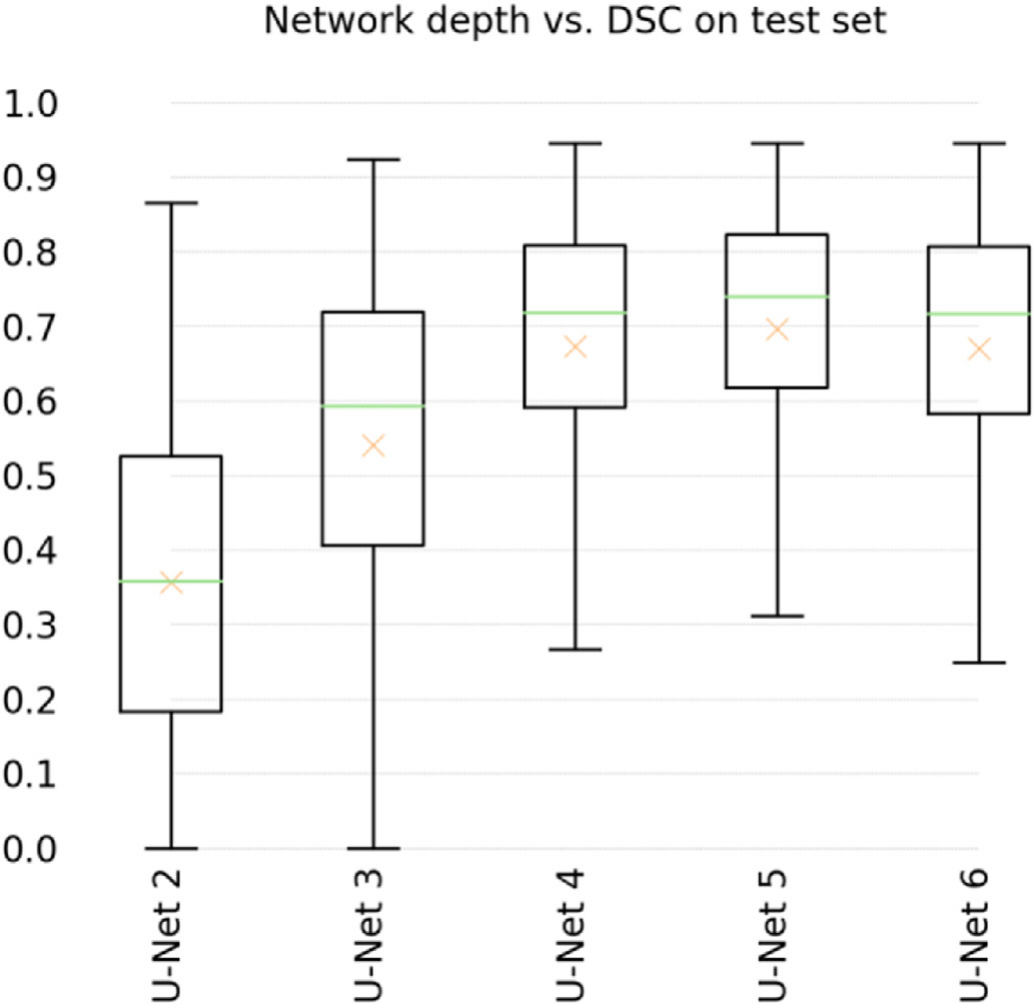
Box-plot of DSC on test set using all evaluated network depths. The crosses additionally represent means.

**Fig. 4:**
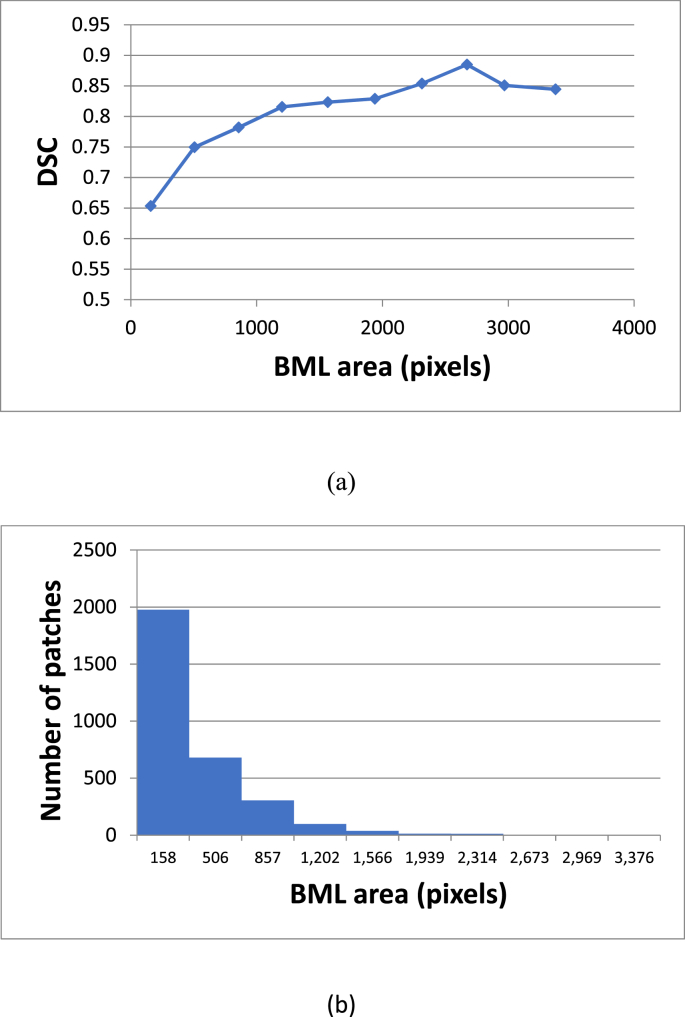
(a) Plot of the average DSC versus BML area in pixels and (b) a histogram of the BML areas in pixels for all 3128 patches. Combined, these plots demonstrate how the large number of small BMLs reduces the overall average DSC value.

A U-Net with 5 levels (U-NET 5) provided the best performing algorithm (Average DSC ​= ​0.70) with a 3% margin over both U-Net 4 and U-Net 6, and the lowest standard deviation of all models (Table 1 and Fig. 3). To gain better insight on interpreting the DSC values, Fig. 4 provides a plot of the average DSC as a function of the BML area and a histogram of BML areas. As would be expected, the average DSC is lower for smaller areas where a minor mismatch can cause a greater percent difference in the overlapping regions.

**Table 1:** Mean and standard deviation of the Dice coefficients on test set of all evaluated model depths. The last column lists the number of trainable parameters of each model.

	Mean	Std.dev.	# params
U-Net 2	0.36	0.22	100,961
U-Net 3	0.54	0.23	465,953
U-Net 4	0.67	0.19	1,925,025
U-Net 5	0.70	0.17	7,759,521
U-Net 6	0.67	0.19	26,702,497

**Fig. 5:**
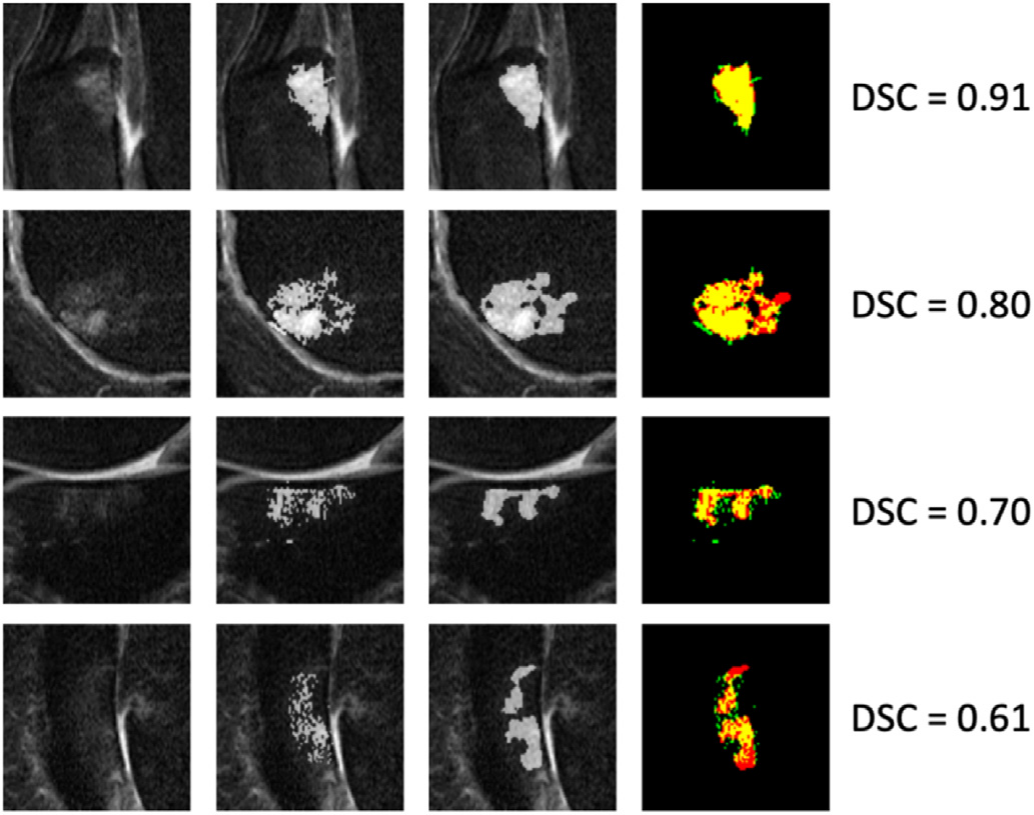
Example BML patches and their predicted segmentation masks. Left to right: image patch, ground-truth, segmentation result, overlay. Overlap is yellow, false positives green, false negatives red. (For interpretation of the references to colour in this figure legend, the reader is referred to the Web version of this article.)

A visualization of the DSC values and the segmented results are shown in Fig. 5, which depicts typical segmentation results over a range of DSC values. The images in the second row shows an example where reader error (green pixels corresponding to the adjacent cartilage) was actually corrected by the CNN algorithm. The results from the 25 scan reliability analysis gave Pearson's R^2^ of 0.95, 0.81, and 0.85 for DL vs. Reader 1, DL vs. Reader 2, and Reader 2 vs. Reader 1 respectively. This showed similar correlation between human readers compared to DL, and the inter-reader agreement.

## 4 Discussion

We offer strong evidence that we have achieved our main goal, which was to obviate the need for the second reader and substantially increase the efficiency of the method. To our knowledge, this is the first published method applying deep learning algorithms to the segmentation of BMLs, although there has been work using DL to grade BMLs according to semi-quantitative scoring [26]. Fig. 2 demonstrates a strong correlation between BML areas from lesions segmented by the trained reader and the DL algorithm. Furthermore, the reader reliability results also show that the DL algorithm can replace a reader without loss of performance. Going forward, we expect to assess large numbers of OAI scans and other data to support new hypothesis-based studies of KOA.

At first glance, an average DCS of 0.7 may not compare favorably to results from studies describing different segmentation tasks. However, there are unique aspects to BML measurement that lead to a lower DSC. First, the BMLs do not have clear and distinctive margins, rather they are diffuse structures, which the DL algorithm has difficulty capturing as exemplified in Fig. 5. More importantly, as demonstrated in Fig. 4, many BMLs are small encompassing just a few hundred pixels or less and DCS values are lower, on average, for small regions. A comparatively high R^2^ value of 0.94 is not surprising since correlation coefficients are highly dependent on the range of values, which for this analysis is large. Probably the most relevant metrics to establish performance and confirm our hypothesis are the inter/intra reader results.

Various network depths were evaluated to find the best model, and a simulation was performed to test the best model in the presence of realistic uncertainty involved in marking the BML locations in image slices. We have shown that the proposed U-Net is good at generalizing even in the presence of imperfections in the training data set, including leakage in segmentation masks. The field of DL continues to evolve with improvements to the software packages, CNN models, and associated hardware. Future approaches leveraging these advances may potentially yield a superior technique.

Our results show that the performance of deeper models is significantly better than shallower models. However, the number of trainable parameters (listed in Table 1) increases geometrically as a function of network depth, suggesting that the lower performance of the deepest model (U-Net 6) in our experiments may be due to overfitting. U-Net 6 has almost four times the number of trainable parameters of U-Net 5, and we hypothesize that the bias-variance trade-off might be most favorable for U-Net 5 in the specific problem addressed here. In general, it is preferable to have the most compact model possible in order to reduce the chances of overfitting, and to keep computational costs low during training and inference.

For future studies of knee OA, it is important to consider workflow. Although the CNN method is substantially faster, a human reader is still required to mark each BML. By eliminating the second reader's step, our CNN approach reduces the time needed to perform analysis of BML volume by approximately 90%. This method also has the potential to be used for studies to predict the incidence and or progression of KOA and as a potentially secondary outcome measure for clinical trials of therapies.

The method is based on the U-Net convolutional neural network architecture, and as such is general enough to be readily extended to other relevant structures in the knee, including cartilage, osteophytes, and effusion synovitis, for which we have developed similar semi-automated methods [10,27,28]. For our current model, the CNN analysis was performed on a slice-by-slice basis not taking advantage ot the three-dimensional nature of MRI data. In the future, we will take the current approach from an assessment of two-dimensional slices to leveraging the three-dimensional nature of MRI, exploring possibilities to replace the BML detection stage in order to fully automate the method. Our method could potentially be used in the clinic with the possibility of integrating the software into a digital radiography product. Combined with other structural measures of KOA, the assessment tool could be used to offer a single measurement of KOA progression probability similar to the FRAX method used to predict fracture for patients with osteoporosis [29].

Our study has several limitations. Although DL improvements are significant, the method is still not fully automated. However, a reader time of approximately 30 ​s per scan will allow for the assessment of thousands of scans in a relatively short amount of time. Perfect agreement is not possible or even necessarily desirable since expert reader variation also occurs as we have demonstrated. We have limited the training and testing to a single MRI standardized protocol from a single study. Further validation and training using different MRI pulse sequences will be necessary to increase generalizability. Finally, our study does little to advance the field of DL and machine learning in general since the algorithmic approach we choose was relatively conventional. Our focus was to document the performance of a highly useful tool that can be used in future clinical studies of KOA and not to make significance advances in computer science.

In conclusion, we developed a novel deep learning-based algorithm to automatically segment BMLs on knee MRI scans. This has the potential to be used in high-powered studies of osteoarthritis, and sets the stage for future developments in the field of OA research.

## Author contributions

Frank Preiswerk: Conceptualization and design, Methodology, Formal Analysis, Investigation, Data curation, Funding acquisition, Writing – original Draft, Writing-review and editing, Visualization, Final approval of the version to be submitted. Meera S. Sury: Conceptualization, Investigation, Acquisition of data, Methodology, Analysis and interpretation of data, Technical support, Writing-review and editing, Final approval of the version to be submitted. Gesa Neumann: Conceptualization, Investigation, Acquisition of data, Methodology, Analysis and interpretation of data, Technical support, Writing-review and editing, Final approval of the version to be submitted. William Wells: Conceptualization, Investigation, Methodology, Analysis and interpretation of data, Technical support, Writing-review and editing, Final approval of the version to be submitted. Jeffrey Duryea: Conceptualization, Methodology, Formal analysis and interpretation of data, Statistics, Investigation, Resources, Writing – review and writing, Project administration and Supervision, Funding acquisition, Final approval of the version to be submitted.

## Declaration of competing interest

The authors declare that they have no conflict of interest.

## References

[1] Felson D.T. (1988). Epidemiology of hip and knee osteoarthritis. Epidemiol. Rev..

[2] Lawrence R.C., Felson D.T., Helmick C.G., Arnold L.M., Choi H., Deyo R.A. (2008). Estimates of the prevalence of arthritis and other rheumatic conditions in the United States. Part II. Arthritis Rheum..

[3] Conaghan P.G., Hunter D.J., Maillefert J.F., Reichmann W.M., Losina E. (2011). Summary and recommendations of the OARSI FDA osteoarthritis assessment of structural change working group. Osteoarthritis Cartilage.

[4] Karsdal M.A., Michaelis M., Ladel C., Siebuhr A.S., Bihlet A.R., Andersen J.R. (2016). Disease-modifying treatments for osteoarthritis (DMOADs) of the knee and hip: lessons learned from failures and opportunities for the future. Osteoarthritis Cartilage.

[5] HCUPnet (2020). https://hcupnet.ahrq.gov/(checked%205/2020).

[6] Mathiessen A., Cimmino M.A., Hammer H.B., Haugen I.K., Iagnocco A., Conaghan P.G. (2016). Imaging of osteoarthritis (OA): what is new?. Best Pract. Res. Clin. Rheumatol..

[7] Segal N.A., Frick E., Duryea J., Nevitt M.C., Niu J., Torner J.C. (2016). Comparison of tibiofemoral joint space width measurements from standing CT and fixed flexion radiography. J. Orthop. Res..

[8] Hunter D.J., Lo G.H., Gale D., Grainger A.J., Guermazi A., Conaghan P.G. (2008). The reliability of a new scoring system for knee osteoarthritis MRI and the validity of bone marrow lesion assessment: BLOKS (Boston Leeds Osteoarthritis Knee Score). Ann. Rheum. Dis..

[9] Ratzlaff C., Guermazi A., Collins J., Katz J.N., Losina E., Vanwyngaarden C. (2013). A rapid, novel method of volumetric assessment of MRI-detected subchondral bone marrow lesions in knee osteoarthritis. Osteoarthritis Cartilage.

[10] Duryea J., Iranpour-Boroujeni T., Collins J.E., Vanwynngaarden C., Guermazi A., Katz J.N. (2014). Local area cartilage segmentation: a semiautomated novel method of measuring cartilage loss in knee osteoarthritis. Arthritis Care Res..

[11] Eckstein F., Cicuttini F., Raynauld J.P., Waterton J.C., Peterfy C. (2006). Magnetic resonance imaging (MRI) of articular cartilage in knee osteoarthritis (OA): morphological assessment. Osteoarthritis Cartilage.

[12] Roemer F.W., Khrad H., Hayashi D., Jara H., Ozonoff A., Fotinos-Hoyer A.K. (2010). Volumetric and semiquantitative assessment of MRI-detected subchondral bone marrow lesions in knee osteoarthritis: a comparison of contrast-enhanced and non-enhanced imaging. Osteoarthritis Cartilage.

[13] Felson D.T., Niu J., Guermazi A., Roemer F., Aliabadi P., Clancy M. (2007). Correlation of the development of knee pain with enlarging bone marrow lesions on magnetic resonance imaging. Arthritis Rheum..

[14] Felson D.T., Parkes M.J., Marjanovic E.J., Callaghan M., Gait A., Cootes T. (2012). Bone marrow lesions in knee osteoarthritis change in 6-12 weeks. Osteoarthritis Cartilage.

[15] LeCun Y., Bengio Y., Hinton G. (2015). Deep learning. Nature.

[16] Liu F., Zhou Z., Jang H., Samsonov A., Zhao G., Kijowski R. (2018). Deep convolutional neural network and 3D deformable approach for tissue segmentation in musculoskeletal magnetic resonance imaging. Magn. Reson. Med..

[17] Norman B., Pedoia V., Majumdar S. (2018). Use of 2D U-Net convolutional neural networks for automated cartilage and meniscus segmentation of knee MR imaging data to determine relaxometry and morphometry. Radiology.

[18] Schneider E., NessAiver M., White D., Purdy D., Martin L., Fanella L. (2008). The osteoarthritis initiative (OAI) magnetic resonance imaging quality assurance methods and results. Osteoarthritis Cartilage.

[19] Collins J.E., Losina E., Nevitt M.C., Roemer F.W., Guermazi A., Lynch J.A. (2016). Semi-quantitative imaging biomarkers of knee osteoarthritis progression: data from the FNIH OA biomarkers consortium. Arthritis Rheumatol..

[20] Ronneberger O., Fischer P., Brox T., U-net (2015). International Conference on Medical Image Computing and Computer-Assisted Intervention.

[21] Long J., Shelhamer E., Darrell T. (2015). Proceedings of the IEEE Conference on Computer Vision and Pattern Recognition.

[22] Dice L.R. (1945). Measures of the amount of ecologic association between species. Ecology.

[23] D. Kinga, J. Ba. Adam: A method for stochastic optimization. International Conference on Learning Representations (ICLR). , vol. 52015.

[24] Chollet F. (2015). https://keras.io.

[25] (Oct, 2018). https://www.tensorflow.org/about/bib.

[26] Astuto B., Flament I., Namiri N.K., Shah R., Bharadwaj U., Link T.M. (2021). Erratum: automatic deep learning–assisted detection and grading of abnormalities in knee MRI studies. Radiology: Artif. Intell..

[27] Hakky M., Jarraya M., Ratzlaff C., Guermazi A., Duryea J. (2015). Validity and responsiveness of a new measure of knee osteophytes for osteoarthritis studies: data from the osteoarthritis initiative. Osteoarthritis Cartilage.

[28] Smith S.E., Hosseinzadeh S., Maetani T., Shilpa P., Collins J.E., Kwoh C.K. (2021). Association of quantitative measures of effusion-synovitis and hoffa-synovitis with radiographic and pain progression: data from the FNIH OA biomarkers consortium. Osteoarthr. Cartil. Open.

[29] Kanis J., McCloskey E., Johansson H., Oden A., Ström O., Borgström F. (2010). Development and use of FRAX® in osteoporosis. Osteoporos. Int..

